# The Dead Can Nurture: Novel Insights into the Function of Dead Organs Enclosing Embryos

**DOI:** 10.3390/ijms19082455

**Published:** 2018-08-19

**Authors:** Buzi Raviv, James Godwin, Gila Granot, Gideon Grafi

**Affiliations:** French Associates Institute for Agriculture and Biotechnology of Drylands, Blaustein Institutes for Desert Research, Ben-Gurion University of the Negev, Midreshet Ben Gurion 84990, Israel; buziraviv@gmail.com (B.R.); godwin.j93@gmail.com (J.G.); granotg@exchange.bgu.ac.il (G.G.)

**Keywords:** dead organs enclosing embryos (DOEE), long-term storage, seed coats, pericarps, glumes, lemmas, paleas, hydrolases, ROS detoxification, cell wall modification, seed microenvironment, seed longevity, seed germination, seedling vigor

## Abstract

Plants have evolved a variety of dispersal units whereby the embryo is enclosed by various dead protective layers derived from maternal organs of the reproductive system including seed coats (integuments), pericarps (ovary wall, e.g., indehiscent dry fruits) as well as floral bracts (e.g., glumes) in grasses. Commonly, dead organs enclosing embryos (DOEEs) are assumed to provide a physical shield for embryo protection and means for dispersal in the ecosystem. In this review article, we highlight recent studies showing that DOEEs of various species across families also have the capability for long-term storage of various substances including active proteins (hydrolases and ROS detoxifying enzymes), nutrients and metabolites that have the potential to support the embryo during storage in the soil and assist in germination and seedling establishment. We discuss a possible role for DOEEs as natural coatings capable of “engineering” the seed microenvironment for the benefit of the embryo, the seedling and the growing plant.

## 1. Introduction

**Figure 1:**
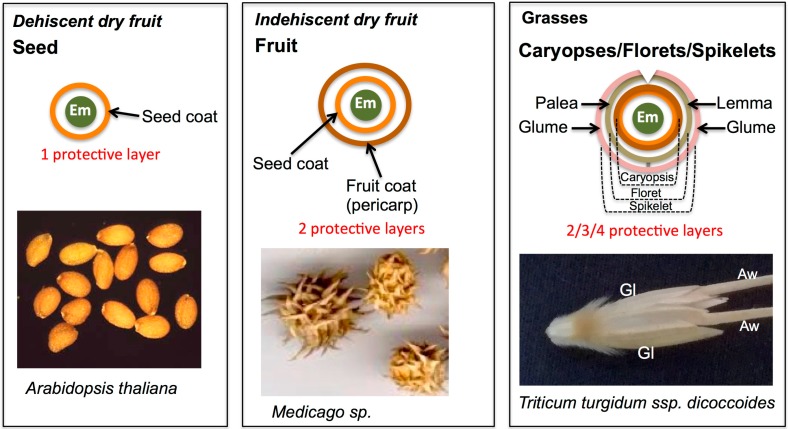
Dispersal units of dry fruits and grasses. Dry fruits can be either dehiscent, in which the fruit is opened at maturity to release the dispersal units, the seeds (e.g., *Arabidopsis thaliana*), or indehiscent, whereby the fruit is not opened at maturity and represents the dispersal unit (e.g., *Medicago* species). In seed and fruit dispersal units, the embryo is covered by one and two protective layers, respectively. In grasses, the basic dispersal unit constitutes a unique type of dry fruit in which the seed coat and the pericarp are fused together to form the caryopsis. A floret is a type of dispersal unit, whereby the caryopsis is covered by the lemma and palea. In a spikelet, a floret or florets are further enclosed by glumes (e.g., *Triticum turgidum* ssp. *dicoccoides*). Note, in caryopsis, floret and spikelet, the embryo is enclosed by two, three and four protective layers, respectively. Em, embryo; Gl, glume; Aw, awn (a long appendage at the lemma).

The seed is the fundamental unit of dispersal in higher plants and is at the focal point of consumers, farmers, seed companies and seed banks. However, plants possess a variety of dispersal units in which the embryo is covered by several layers that provide physical protection and means for dispersal [1,2]. Dry fruits consist of two groups: dehiscent, in which the fruit is splitting open at maturity to allow for seed dispersal, and indehiscent, whereby the fruit is not opened at maturity and constitutes the dispersal unit (Figure 1). Consequently, seeds, the dispersal unit of dry dehiscent fruits, have a single protective layer (the seed coat) enclosing the embryo while in fruits the embryo is covered by two protective layers, the seed coat and the fruit coat (the pericarp and its accessories). In various Poaceae species, the basic dispersal unit constitutes of a unique type of dry fruit in which the seed coat and the pericarp are fused together to form the caryopsis. Two additional types of dispersal units are common in Poaceae species: a floret, in which the caryopsis is covered by the lemma and palea, and a spikelet, whereby the floret is further covered by the glumes (Figure 1).

All protective layers enclosing the embryo, namely, seed coats, pericarps, lemmas, paleas and glumes, are maternally-derived and undergo programmed cell death (PCD) at maturity. Although the term dispersal unit highlights an entity that is specialized for dispersal, several studies have shown that the dispersal unit serves multiple functions including protection from predation, seed positioning in the soil, moisture adsorption, seed anchoring, light filtering, and regulation of seed respiration [3]. The mechanisms of seed protection from predation include morphological characteristics such as hairiness, thickness, and hardness of seed coat as well as chemical protection, in which the dispersal unit contains secondary metabolites that control predation [4,5]. Sometimes the covering layers of the dispersal unit contribute to dormancy and/or inhibit germination due to permeability barriers preventing water uptake or gaseous exchange, the presence of germination inhibitory substances [6,7,8,9,10] or due to mechanical barriers preventing embryo expansion [11]. The composition and level of substances within the dead organs enclosing embryo (DOEEs) may be affected by environmental cues. For instance, the pericarps of pea (*Pisum sativum*) possess nutritional and antioxidant compounds that were further enhanced by plant growth promoting microbes [12]. Several reports demonstrated the positive effect of the intact dispersal units on seed longevity and seedling establishment when compared to naked seeds [13,14,15,16].

It is commonly believed that during PCD most macromolecules such as DNA, RNA and proteins are degraded and their constituents remobilized into other plant parts [17,18,19]. Contrary to this view, recent reports have demonstrated the capacity of DOEEs to store and maintain the integrity of hundreds of proteins; some can persist in active forms for decades, and are released to the immediate surrounding of the dispersal unit upon hydration [16,20,21].

In this review, we highlight recent studies showing that DOEEs of various species across families have the capability for long-term storage of various substances including active proteins (hydrolases and Reactive Oxygen Species (ROS) detoxification enzymes), nutrients and metabolites that have the potential to support the embryo during storage in the soil and assist in germination and seedling establishment. We discuss the potential function of DOEEs as natural coatings, acting as engineers of the seed microenvironment to increase survival rate of the seed.

## 2. DOEEs Release Hundreds of Proteins upon Hydration

Proteome analyses of DOEEs including seed coats, pericarps and glumes highlighted their function as a long-term storage for hundreds of proteins that are released upon hydration. Many of the identified proteins possess catalytic activity including hydrolytic activity and oxireductase activity; some of the stored proteins, such as nucleases, remain active after long periods (10–50 years) of storage within DOEEs [16,20,21]. Proteins released from DOEEs include several plant defensin-like (DEFL) molecules, also known as low molecular weight cysteine-rich (LCR) proteins, which are implicated in defense response to fungus [22,23,24]. These proteins, primarily found in seeds, are also present in leaves and flowers and often up-regulated following pathogenic attack or in response to environmental stress such as drought [25]. They can confer enhanced resistance to pathogen when overexpressed in transgenic plants [26,27]. Other proteins identified in the proteome data that could act against pathogens include chitinases, endochitinases, endonucleases and glucanases. Chitinases degrade chitin, a polysaccharide found in a variety of organisms including insects and fungi. Chitinase and glucanase genes are often over-expressed in plants and are implicated in combating fungal pathogens [28,29,30]. The proteome data also revealed S1 type endonucleases, which are released from DOEEs upon hydration [16,20,21]. Endonucleases, in general, are involved in multiple cellular processes including DNA synthesis and DNA repair [31] and in PCD [32,33,34]. The capacity of endonucleases to target unpaired regions within superhelical DNA to introduce nicks and double strand DNA breaks may implicate them as defense factors against plasmid-containing soil pathogens such as the *Clavibacter michiganensis* subsp. *michiganensis*, a Solanaceae species-pathogenic actinomycete that contains two plasmids, which are important for pathogenesis [35]. In addition, transgenic tobacco plants expressing a bovine pancreatic RNase, an extracellular ribonuclease, showed an increase resistance to plant RNA viruses, namely, Cucumber mosaic virus and Tobacco mosaic virus [36,37]. Notably, treatment of leaves with S-like RNase NE injected into the extracellular space suppresses growth of *Phytophthora parasitica* [38,39], an oomycete soil borne pathogen with a wide range of host plants. Also, proteome analysis identified Pathogenesis Related 4 (PR4 RNase) homolog ribonucleases released from dead seed coats of white mustard (*Sinapis alba*) and from dead glumes of wild emmer wheat [16,20]. The Wheatwin1 PR4 RNase activity was shown to be required for repressing fungal cells activity [40]. The presence of plant defense-related proteins within the dead, non-living organs of wheat and oat dispersal unit (DU) was reported. Accordingly, wheat bran was found to contain multiple plant defense-related proteins including oxalate oxidase (OXO), peroxidase (POX), and polyphenol oxidase (PPO) whose activities were highest in the outer layer [41]. In addition, when oat DU was incubated with *Fusarium avenaceum* strain *F.a.*1, polyphenol oxidase (PPO) was induced in the whole DU as well as in the dissected parts, namely the dead, non-living hulls (lemma and palea) and the caryopses [42]. The authors suggested that the induction of PPO in the non-living hulls, surprisingly, could have resulted from latent forms of PPO that are activated following challenge with *F.a.*1 [42].

The Proteome data also revealed two groups of proteins released upon hydration from DOEEs that might play an important role in seed persistence in the soil and seed germination, namely, ROS detoxifying enzymes (e.g., superoxide dismutases and peroxidases) and cell wall modification enzymes (e.g., pectinesterases and polygalacturonases) [16,21], which are discussed below.

## 3. ROS Detoxifying Enzymes

ROS have an important role in seed biology [43,44]. They are produced during seed maturation and desiccation, seed storage in the soil and seed germination. ROS could lead to oxidative stress that can harm macromolecules such as proteins and DNA, and consequently to seed weakening. Thus, ROS “detoxifying” enzymes such as superoxide dismutases (SODs), catalases and peroxidases are of prime importance in maintaining appropriate balance of ROS and seed viability. Although ROS have long been thought as toxic molecules, many reported studies highlighted ROS also as signaling molecules acting in releasing seed dormancy, germination as well as providing defense against soil pathogens [43,44,45].

The term ROS “detoxifying” enzymes may be ambiguous, inasmuch as many of the enzymes actually convert one ROS into another often even more potent species. Accordingly, radical derivatives of oxygen such as superoxide (O_2_^−^), resulting from reduction of oxygen, is a short-lived molecule that serves as a precursor for superoxide dismutase leading to the formation of other ROS including hydrogen peroxide (H_2_O_2_) and hydroxyl radical (OH**^·^**). Catalase catalyzes the conversion of H_2_O_2_ into water and oxygen. Alternatively, H_2_O_2_ may react with glutathione peroxidase to catalyze the formation of water and the conversion of reduced glutathione (GSH) into glutathione disulfide (GSSG). The presence of transition metal allows hydroxyl radical production from H_2_O_2_ by the Fenton reaction. Accordingly, Fe^2+^ is oxidized by H_2_O_2_ to Fe^3+^ to form a hydroxyl radical (HO•) and a hydroxide ion (OH^−^) [46].

During early development, many seeds are green and engaged in photosynthesis and thus the production of superoxide and singlet oxygen (^1^O_2_), another type of ROS, is inevitable. Singlet oxygen is a highly reactive oxygen species that mostly reacts with organic molecules having double bonds [47]. Damages imposed by singlet oxygen can reduce photosynthetic efficiency and even cause cell death. Various antioxidant compounds within the chloroplasts such as carotenoids, tocopherols and plastoquinones quench singlet oxygen and protect against its toxic effects [48]. Nevertheless, the cell responses to the presence of singlet oxygen are largely dependent on its levels. Accordingly, extreme production of singlet oxygen might lead to unavoidable death known as “accidental cell death” and moderate levels may induce programmed cell death, while low levels of singlet oxygen may signal for acclimation [48].

An important source for ROS is the mitochondrial respiratory system in which electron leakage from the transport chain can generate superoxide that can be dismutated into H_2_O_2_ [49]. Hence, the amount of ROS generated in the seed is proportional to the mitochondrial respiratory activity being high at early stages of embryogenesis but strongly reduced as seed mature and become quiescent. During imbibition and germination, the respiratory activity is significantly enhanced leading to production of ROS. Accordingly, superoxide and H_2_O_2_ are produced at high levels in embryonic axes of soybean during imbibition, which was associated with increase in activity of ROS enzymes including SODs, catalase, peroxidase, glutathione and ascorbate peroxidases [50]. ROS are released from the seed coat and the embryo of radish seeds upon imbibition in the dark. A correlation was found between inhibition of germination caused by far-red light and inhibition of ROS release; Gibberellic acid (GA) restores full germination under far-red light and the release of ROS from the seeds [51]. Similarly, in barley (*Hordeum vulgare*) aleurone cells, H_2_O_2_ production was induced by GA but suppressed by ABA [52]. Presently, it is not clear whether induction of ROS production during imbibition is a developmentally regulated process that facilitates germination or toxic by-products induced upon resumption of respiration activity during germination. The findings that ROS production is often associated with increase expression of genes encoding for ROS “detoxifying” enzymes suggest that ROS are toxic compounds whose levels must be decreased. Thus, the overrepresentation of ROS detoxifying/metabolizing enzymes in the dead glumes of wild emmer wheat [16,21] highlighted their importance in seed persistence in the soil, germination and seedling establishment. We hypothesize that these enzymes are released upon hydration to the immediate surrounding of the germinating seed to fulfill multiple functions. Accordingly, ROS detoxifying enzymes make sure that the seed microenvironment is free of hazardous radicals or allow for the production of specific radicals, which are important for germination, or are necessary for protection against potential soil pathogens. In this respect, the presence of multiple SOD enzymes in DOEEs might ensure reduction in superoxide level and generation of H_2_O_2_ that facilitates seed germination [53]. Consequently, H_2_O_2_ may be reduced to hydroxyl radical, which in turn might affect seed longevity and seed germination [54,55].

## 4. Cell Wall Modification Enzymes

The overrepresentation of pectinesterases (PMEs/PEs) and polygalacturonases (PGs) in DOEEs [21] suggests a role in modifying cell walls to allow for the radicle to protrude outside the seed coverings. Both PMEs and PGs are pectinases involved in multiple developmental processes in plants via loosening and softening pectin—the major constituent of plant cell walls. PMEs and PGs were reported to affect the mechanical stability of cell walls during fruit ripening. They are involved in cell wall loosening of the endosperm and the testa, which is necessary for radicle protrusion, cell wall extension during pollen germination and pollen tube growth, abscission and stem elongation [56,57].

PMEs are responsible for de-methyl esterfication of the most abundant pectin in cell walls, homoglacturonans (HG), which in turn affects their elasticity, permeability and porosity and consequently plant growth and development [58,59]. The removal of methyl groups from HG by PMEs can differently affect cell wall properties leading either to cell wall softening or stiffening. Notably, the activity of PMEs is subjected to regulation by pectin methylesterase inhibitors (PMEIs), a group of small proteins that physically interact with PMEs and affect their activity [60]. PMEs were isolated from germinating seeds of a variety of annual and perennial plant species including cowpea (*Vigna sinensis*) and yellow cedar (*Chamaecyparis nootkatensis*) and are assumed to play an important role in loosening cell walls to allow for radicle emergence [61,62]. Changes in expression pattern of PME enconding genes as well as in PME enzymatic activities observed during seed germination of *Lepidium sativum* (garden cress) are associated with testa rupture; exogenous application of PMEs to garden cress seeds promoted permeability and rupture of the testa [63]. Transgenic *Arabidopsis* plants overexpressing PME inhibitor (*PMEI5*) displayed, as expected, reduction in PME activities and increased cell wall methylesterification, which was accompanied by a significantly faster rate of germination [64]. Closer inspection of published data [64] revealed that most methylesterification, as deduced by JIM7 immunolabeling, was found in embronic cells, with no clear effect on methylesterification of the testa. It appears that the action of PMEs is complex and that demethylesterification may be required, in a temporal manner, to coordinate between the growing radicle and the rupture of the endosperm and the testa.

Polygalacturonases (PGs) are enzymes that hydrolyze the α-1,4 glycosidic bonds between galacturonic acid residues resulting in pectin depolymerization and softening of cell walls. A calcium-dependent exo-PG activity was detected in tomato seed protein extracts, which is related to the product of the *LeXPG1* gene. Indeed, the LeXPG1 mRNA was increased during imbibition, and further enhanced in seeds upon completion of germination, suggesting that PG is involved in weakening of the endosperm cell walls for radicle protrusion [65]. Similarly, PG was reported to be involved in lateral root development in *Allium porrum* via loosening of cortical cells ahead of the growing tip of the root [66].

Taken together, PMEs and PGs, which are accumulated in DOEEs provide a complementary, maternally source for cell wall modifying enzymes to ensure proper seed germination by loosening seed coverings at the time of germination, to allow for radicle protrusion.

## 5. DOEEs as a Rich Storage for Nutrients and Growth Factors 

The study of the nutritional value of seed coats, pericarps and glumes revealed that high levels of nutrients such as potassium, phosphorus and sulfur are stored within these dead organs and are likely to serve as an immediate nutritional supply for germinating seeds. Germination assays of wild emmer wheat showed the beneficial effect of the intact dispersal unit (DU) compared to naked caryopsis. Although germination from the DU was delayed by 4–5 days, post germination growth and development were enhanced in seedlings derived from the intact DU. Particularly, DU-seedlings have significantly higher number and higher length of lateral roots than seedlings derived from naked caryopsis [16], which are attributed to the effect of lateral root-promoting substances such as auxin [67,68,69] and to nutritional elements [70,71,72,73], respectively. Similar effects were reported for germination of the Winterfat (*Krascheninnikovia lanata*) dispersal unit, which consists of hairy bracts enveloping utricle (fruit) and the seed. Accordingly, the removal of the hairy bracts significantly reduced seedling establishment and vigor [13]. Notably, initial analysis of phytohormones in glumes derived from wild emmer wheat revealed the presence of abscisic acid (ABA) and auxin (IAA); interestingly, jasmonic acid (JA) and salicylic acid (SA) were most abundant in the glumes [74]. The significant of these phytohormones in seedling growth and development as well as in defense priming against biotic and abiotic stresses still need to be explored.

Potassium is one of the major essential nutrients for plant growth and development. The high levels of potassium identified in DOEEs might reflect the accumulation of large quantities of this element in plants, which constitutes 2–10% of plant dry weight [75,76]. Potassium has important regulatory roles, and is required for plant growth processes including enzyme activation, photosynthesis and protein synthesis; it also participates in plant response to biotic and abiotic stresses [77].

## 6. Control of Microbial Growth by DOEEs

Seeds are commonly germinated in the soil, whereby radicles (embryonic roots) are protruding into an unknown, potentially hazardous environment, which challenge their survival. Even before germination, during storage in the soil, seeds are subjected to biotic and abiotic stress conditions (microbial attack and humidity fluctuations), which might affect their longevity and persistence; however, seeds in the soil maintain viability for many years [78]. The mechanisms underlying long-term viability of seeds in the soil were addressed mainly with respect to chemical defense (secondary metabolites). Accordingly, examination of seeds of over 80 plant species from the British flora revealed that many possess and release upon hydration potent antimicrobial and antifeedant compounds including hydroxyphenols and hydrogen cyanide [79]. Seeds of various plant species secrete, following hydration, various proteins that function against fungal pathogens and Gram-positive bacteria [26,80,81], but their origin, maternal (e.g., dead seed coat) or zygotic (i.e., embryo) is not well known. Many seeds have pigmented testa resulting from production of phenolic compounds (e.g., tannins), which are often associated with defense activity against pathogens [82,83,84]. In addition, proteome analysis of soybean (*Glycine max* L.) live seed coats derived from developing seeds showed multiple proteins including an abundant class I chitinase [85] that plays a role in plant defense against pathogens [86,87].

Recent data showed that dead seed coats contain microbial growth promoting or suppressing activities. Analysis of seeds derived from two cruciferous species, namely, *Sinapis alba* and *Anastatica hierochuntica*, showed that they differ in their bacterial growth controlling activities. Substances secreted from *A. hierochuntica* embryonic tissues or seed coats displayed very strong antibacterial activity toward Gram-positive and Gram-negative bacterial strains *Staphylococcus aureus* and *Escherichia coli*, respectively, which was comparable to the inhibitory effect of 100 μg/L ampicillin [20]. Substances secreted from *A. hierochntica* seeds also displayed strong inhibitory effect on spore germination of *Fusarium oxysporum* f.sp. *melonis* [20]. On the other hand, substances released from *S. alba* seeds had no antibacterial activity but rather slight bacterial growth promoting activity. These differences may be attributed to the different habitats from which seeds were collected. Thus, *S. alba* seeds were collected from plants growing along the margins of agricultural fields in the northern Negev and supplemented with all intensive agricultural treatments including irrigation, fertilization and chemicals against pests and bacterial and fungal pathogens. Seeds of *A. hierochuntica*, on the other hand, were collected from natural habitat in the Negev desert and mother plants were subjected to all kinds of stresses prevailing in the desert ecosystem. Thus, while substances secreted from *A. hierochuntica* might combat potential soil pathogen, those secreted from *S. alba* seeds might promote growth of beneficial soil borne microbes to support plant growth and development. Plant growth-promoting bacteria can influence root development and enhance plant growth by various means including increasing nutrient availability (e.g., nitrogen (N), iron (Fe) and phosphorus (P)) and production of indolic compounds (e.g., IAA) as well as by protecting plants from diseases, at least partly by suppressing deleterious soil borne pathogens [88,89,90]. Thus, accumulation of microbe growth controlling substances in DOEEs might act directly or indirectly to control soil borne pathogens and to promote plant growth and development.

## 7. Concluding Remarks

**Figure 2:**
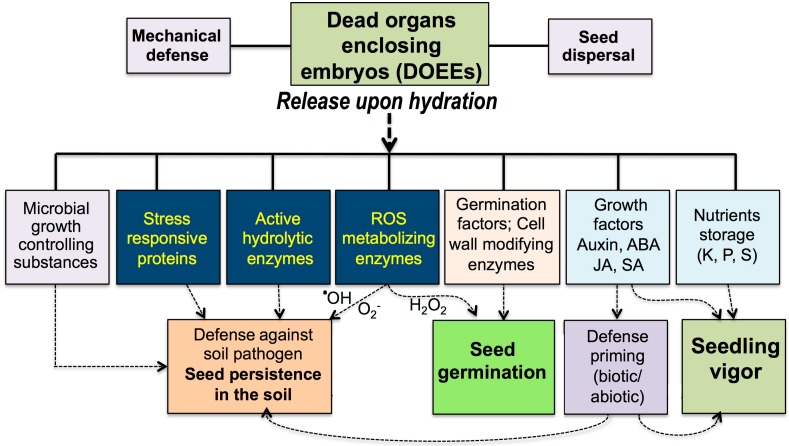
Dead organs enclosing embryos (DOEEs): More than a physical shield for embryo protection and means for seed dispersal, DOEEs function as a rich, long-term storage for multiple beneficial substances that are released upon hydration to the immediate surroundings of the dispersal unit (DU) including seeds, indehiscent dry fruits, florets and spikelets. These substances comprise active proteins (hydrolases, ROS metabolizing enzymes, etc.), metabolites (e.g., phytohormones) and nutrients that are released from DOEEs (e.g., seed coat, pericarp, and glumes) and have the potential to facilitate germination, confer defense against soil pathogen, trigger defense priming in germinating seeds toward biotic and abiotic stresses and supply nutrients and growth factors that contribute to seedling establishment and vigor.

DOEEs, including seed coats, pericarps and floral bracts, in grasses were evolved not just for providing a physical shield for embryo protection or means for seed dispersal and germination but also as storage organs for multiple active proteins and probably metabolites and other substances for the purpose of germination, nourishment as well as protection of germinating seeds from soil pathogens (Figure 2). Thus, our data suggest that DOEEs should be viewed as “natural coatings” capable of “engineering the microenvironment” to allow for seed persistence in the soil, germination and seedling establishment. These findings open a new realm of research in seed biology and raised several questions for future study:

1. Do substances released from DOEEs (e.g., JA, SA) have the capability of inducing plant defense priming against biotic and abiotic stresses?
2. Can we use substances released from DOEEs as a substitute for the hazardous chemical coating of seeds?
3. Does storage of seeds in gene banks with their associated dead organs better preserve and maintain seed viability?
4. How do mother plant growth conditions affect the composition of substances stored in DOEEs and consequently seed longevity, germination and seedling establishment?
5. Can we modify the composition of proteins and of other substances within DOEEs to build up a superior natural coating?

Taken together, the way we commonly refer to death in plants is challenged by the findings emerging from the study of DOEEs. The findings that the “dead can nurture” add another dimension, not recognized previously, to understanding seed biology and ecology, and might have important implications for seed economy and for ex-situ conservation of seeds in gene banks for future usage.
